# Artificial Intelligence Applications in Emergency Toxicology: Advancements and Challenges

**DOI:** 10.2196/73121

**Published:** 2025-08-22

**Authors:** Lorraine Pei Xian Yong, Joshua Yi Min Tung, Nicole Mun Teng Cheung, Zi Yao Lee, Ee Yang Ng, Alexander Jet Yue Ng, Clement Kee Woon Lim, Yuru Boon, Daniel Yan Zheng Lim, Gerald Gui Ren Sng, Jonathan Zhe Ying Tang

**Affiliations:** 1 Urgent Care Centre, Alexandra Hospital, Singapore, Singapore; 2 Emergency Medicine Department, National University Hospital, 5 Lower Kent Ridge Road, Singapore, 119074, Singapore, 65 67725000; 3 Data Science and Artificial Intelligence Laboratory, Singapore General Hospital, Singapore, Singapore

**Keywords:** artificial intelligence, toxicology, AI, clinical toxicology, emergency toxicology

## Abstract

Emergency toxicology is a complex field requiring rapid and precise decision-making to manage acute poisonings effectively. Toxic exposures are often unpredictable, and the constraints of time and resources often challenge conventional diagnostic and treatment approaches. Artificial intelligence (AI) has emerged as a valuable tool in emergency medicine, offering the potential to enhance diagnostic accuracy, predict clinical outcomes and improve clinical decision support systems. Despite the increasing focus of AI in medicine, its applications in emergency toxicology are still underexplored. This viewpoint aims to provide perspectives on AI applications in emergency toxicology by highlighting key advancements, challenges, and future directions. While AI has demonstrated significant potential in improving toxicological predictions through various applications, challenges such as data quality, regulatory concerns, and implementation barriers are still hurdles to its use. Further research, regulatory frameworks, and integration strategies are needed to ensure effective and ethical implementation in clinical practice.

## Introduction

Acute poisonings and chronic exposure to chemicals represent a significant global health care burden, with an estimated 2 million lives and 53 million disability-adjusted life-years lost in 2019 [1]. Poisoning is a major cause of emergency department visits globally, and studies have shown that children and young adults are commonly affected [2,3]. Data from the United States Poison Control Centers reflect a concerning trend of rising numbers of severe toxic exposures leading to more serious outcomes [4]. Emergency care providers operate in a high-stakes environment where they are constantly challenged by the vast and everchanging landscape of toxic exposures. These exposures can originate from a variety of sources, including prescription and over-the-counter pharmaceuticals; illicit drugs; industrial chemicals used in manufacturing and other industries; household products such as cleaning agents, pesticides, and paints; and natural toxins. The sheer diversity of potential toxins, coupled with the often-unpredictable nature of their effects, makes the diagnosis and treatment of toxic exposures a particularly demanding aspect of emergency medicine.

A detailed risk assessment—considering the agent, dose, time of ingestion, route of exposure, drug formulation, and the individual’s underlying health conditions—is important to aid management decisions. Poisoning symptoms are often nonspecific, and the window for decontamination and targeted antidotal therapy is narrow, necessitating rapid diagnosis and intervention. However, the clinical variability of such ‘toxidromes’ can further complicate the diagnostic process and delay the initiation of appropriate treatment, leading to morbidity or mortality. Emergency care providers must therefore possess a broad knowledge base and be able to quickly assess and manage patients with a wide range of toxic exposures, often with limited information and under significant time pressure. In addition to the challenges posed by the diversity and unpredictability of toxic exposures, other challenges in the field of emergency toxicology include the limited availability of specialized expertise and resources, particularly in rural or under-resourced environments, and the difficulty in obtaining accurate medical histories in a timely manner, especially in cases of unwitnessed exposures or obtundation.

Conventional approaches, while effective in many cases, rely heavily on the clinician’s expertise and access to clinical toxicology consultation or toxicology databases, which can vary widely across health care settings. Artificial intelligence (AI) has emerged as a potential solution in many areas of health care, with proven potential to enhance diagnostic precision, predictive analytics, and clinical decision support systems across a variety of disciplines [5,6]. In emergency toxicology, AI holds the promise for improving and expediting patient care by facilitating the rapid identification of toxins, predicting clinical trajectories, and recommending tailored treatment strategies. By using AI-driven tools such as machine learning (ML) algorithms and natural language processing (NLP), clinicians can address gaps inherent in traditional toxicology workflows, ultimately improving patient outcomes and operational efficiency. This paper explores the applications, challenges, and future directions of AI in emergency medicine toxicology, and its potential to redefine the management of acute poisonings in both high-resource and austere settings.

## AI Applications in Emergency Toxicology

**Figure 1.**
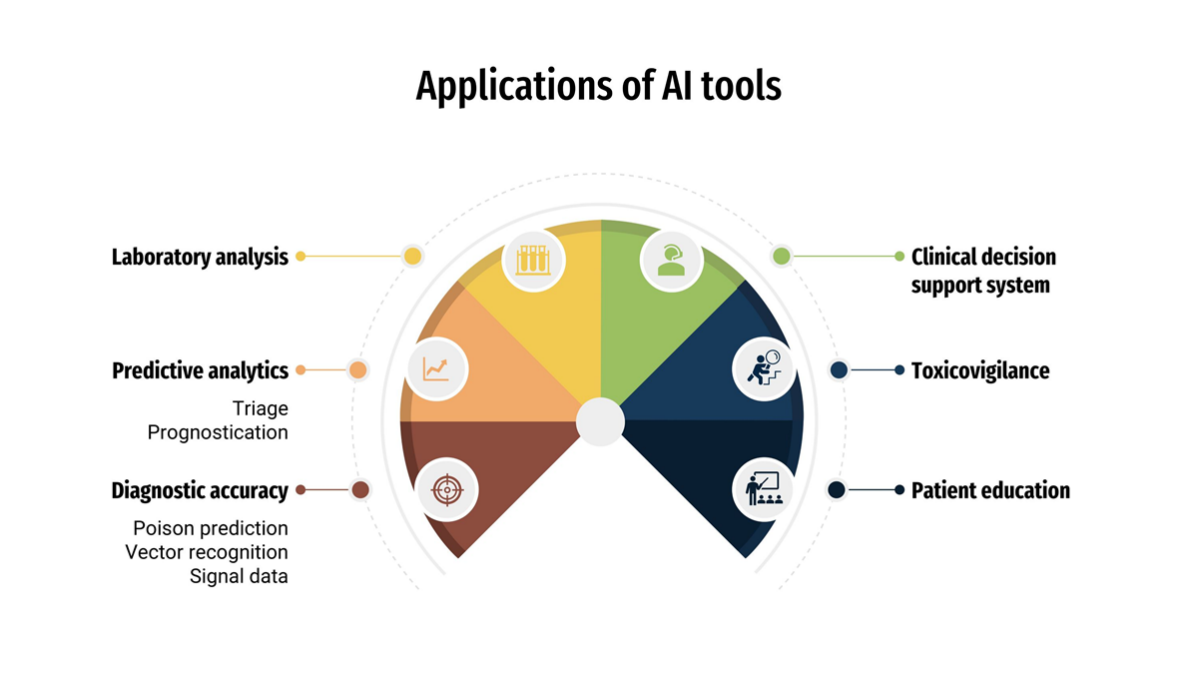
Graphical summary of applications of AI tools.

In the fast-paced environment of emergency medicine, clinicians are often faced with the challenge of managing complex and unpredictable toxic exposures under significant time and resource constraints. AI tools offer the opportunity to enhance diagnostic accuracy, predict outcomes, and guide treatment decision. The following section highlights the diverse applications of AI in emergency toxicology, demonstrating its potential in clinical practice and patient care, as shown in Figure 1.

### Improving Diagnostic Accuracy

#### Poison Prediction

Obtaining accurate exposure history in patients with acute poisoning can be challenging, as patients are often unable to convey a verbal history to attending health care providers. Diagnosis is therefore dependent on a constellation of clinical symptoms (toxidrome-based approach), and the emergency physician’s experience and clinical judgment. AI-based applications have demonstrated capabilities in identifying the causative drug in acutely poisoned patients. Early attempts by Chary et al [7] in using probabilistic logic networks to mimic knowledge representation and decision-making of clinicians in classifying toxidromes showed comparable performance on easy-to-intermediate-difficulty synthetic case scenarios. However, the logic network performed worse when compared against two human medical toxicologists on challenging cases.

More recently, using the United States’s National Poison Data System (NPDS) of 201,031 entries, Mehrpour et al developed machine learning (ML) and deep neural network (DNN)–based models to distinguish between single-agent poisonings with eight drugs: acetaminophen, diphenhydramine, aspirin, calcium channel blockers (CCBs), sulfonylureas, benzodiazepines, bupropion, and lithium. Their ML model demonstrated an overall specificity of >92%, with >99% specificity for sulfonylureas, CCBs, lithium, and aspirin [8]. Meanwhile, the DNN models built with PyTorch and Keras showed specificity of 97% and 98%, respectively [9].

The development of ToxNet by Zellner et al [10] from the Technical University of Munich marks a further advancement in the use of AI-based tools in poison prediction. The ToxNet architecture comprises a literature-matching network and graph convolutional network functioning in parallel, optimized using inductive graph attention networks. This computer-aided diagnosis system, trained using data from 781,278 recorded calls, showed superior performance when compared against other algorithmic models, and, more critically, when compared against clinicians experienced in clinical toxicology.

#### Vision Models for Vector Recognition

AI models have also demonstrated use cases in vector recognition. Vision language models and convolutional neural networks have the capabilities to recognize and classify objects, and have been shown to aid in diagnosis in other medical fields such as dermatology [11]. In the field of emergency toxicology, these new technologies have demonstrated utility in snakebite and toxic plant identification, which has the potential to aid emergency toxicologists immeasurably. In 2019, de Castañeda et al [12] published a commentary in Lancet Digital Health calling for the empowerment of neglected communities and health care providers by embracing the AI revolution for snakebite identification. This call to action was heeded by groups such as Bolon et al [13], who in 2022 developed an AI model based on vision transformer architecture, trained on a dataset of 386,006 snake photos, to identify snakes from across the world. The model achieved an unprecedented macro-averaged *F*_1_-score of 92.2%, with accuracy at the species and genus level of 96.0% and 99.0%, respectively [13]. In 2023, Zhang et al conducted a systematic review of AI use in snakebite identification, concluding that AI–based methods had the capability to quickly and accurately distinguish between venomous and nonvenomous snake species [14].

In 2021, Wagner et al [15] described methods for mushroom data creation and curation to support classification tasks. Such groundwork appears to have borne fruit, as evinced in a 2024 case report on the use of Google’s Gemini AI to accurately identify a toxic plant species (*Datura stramonium*) in a patient presenting to a Aksaray Training and Research Hospital in Turkey with restlessness, altered mental state, and hallucinations that occurred 2 hours after consumption of an herbal tea [16]. No botanist was available at the time, and successful treatment was administered based on Gemini’s identification of the plant seed. Later consultation with a botanist confirmed the identity of the seed in question.

#### Signal Data

Point-of-care tests used in the emergency department, such as electrocardiographs (ECGs), contain a wealth of signal data that may be interpretable by AI models. Chang et al [17] used deep learning (DL) methods to train an AI system to detect digoxin toxicity using ECG data alone. The area under the curve (AUC) of the model in a human-machine comparison test was 0.929, demonstrating noninferiority compared with experienced emergency and cardiovascular staff members and an emergency chief resident.

AI–driven tools are reshaping the diagnostic landscape in emergency toxicology by enhancing the accuracy and efficiency of poison identification and vector recognition. These tools have the capacity to reduce diagnostic uncertainty and expedite decision-making in high-stakes environments, laying the groundwork for more consistent and effective interventions in emergency toxicology cases [8].

### Predictive Analytics

#### Triage

Early and accurate identification of patients at risk of severe outcomes using AI–based predictive models has the potential to change current triage workflows. Moulaei et al [18] compared DL and ML models for prediction of intubation necessity in methanol-poisoned patients using a dataset of 897 cases. Their Long Short-Term Memory (LSTM) model from the DL group, and Random Forest (RF) and Extreme Gradient Boost (XGB) models from the ML group, demonstrated high specificity and sensitivity of up to 99% and 100%, respectively [18].

#### Prognosticating Clinical Outcomes

Similarly, several studies have investigated the utility of AI–based methods to forecast the clinical trajectory of patients with acute poisoning. RF models have yielded high predictive accuracy in carbon monoxide [19], acetaminophen [20], and diquat poisonings [21], whereas other ML methods such as XGB and Support Vector Machine (SVM) have been applied to methadone [22] and metformin poisonings [23]. A summary of studies investigating AI–based models for prognosis in acute poisonings is shown in Multimedia Appendix 1.

### Laboratory Analysis

AI-based tools for laboratory analyses may offer enhanced diagnostic precision in toxicology patients. Chen and Hu from the Wenzhou Medical University investigated the use of SVMs for the prognosis of paraquat-poisoned patients, using arterial blood gas and complete blood count indexes. Using a particle swarm optimization algorithm to optimize SVM parameters, they achieved a maximum accuracy of 76% using arterial blood gas indexes [24]. Separately, using complete blood count indexes yielded an accuracy of 85.2% and a specificity of 95.1% [25].

### Clinical Decision Support Systems

The next step beyond predictive analytics involves the deployment of AI–based models as clinical decision support systems, by integrating datasets and providing evidence-based recommendations. Prior studies in pharmacology have demonstrated the utility of AI models in therapeutic drug monitoring and model-informed precision dosing across a wide range of medication classes [26]. In the field of emergency toxicology, Mohtarami et al [27] developed a XGB model to predict the maintenance dose and duration of administration of naloxone in opioid toxicity cases, achieving an AUC of 0.97 .

### Toxicovigilance

Advancements in NLP and the advent of large language models have opened possibilities for toxicovigilance via monitoring of real-time data from diverse sources such as social media. Sato et al [28] from Keio University used NLP techniques to analyze 30,203 social media posts on Twitter (subsequently rebranded X), to identify trends and patterns in drug misuse, including mentions of overdoses on medications such as codeine and pregabalin. Such monitoring could facilitate early identification of emerging threats, and inform preventive strategies. Shah-Mohammadi and Finklestein [29] applied retrieval-augmented generation models integrated with GPT-4 to improve the extraction of substance use data from clinical notes, flagging patients with risk factors for substance use disorders.

In addition to analyzing social media and clinical data, toxicovigilance efforts have benefited from predictive models aimed at identifying populations at risk of exposure to toxic agents. Potash et al [30] developed an ML model using random forests to predict childhood lead poisoning by analyzing spatiotemporal data, housing characteristics, and blood lead level surveillance data, showing superior predictive performance compared to regression modeling [30]. Such targeted prediction facilitates the allocation of public health resources, enabling proactive rather than purely reactive interventions.

### Patient Education

Large language models are a form of AI pretrained on a vast corpus of text, image, or video data. These generative AI models such as ChatGPT are capable of producing human-like text and visual output and have demonstrated efficacy in providing clear and accurate explanations regarding poisoning symptoms, treatments, and preventive measures. In studies comparing AI-generated content to that of clinical toxicologists, ChatGPT’s responses were often indistinguishable from expert-generated content and were rated highly for readability and relevance [31,32]. The flexibility of generative AI-based systems allows for the tailoring of educational materials to individual needs, potentially improving health literacy and empowering patients to make informed decisions.

## Limitations and Challenges

### Data Quality and Bias

One of the most significant challenges in using AI in emergency toxicology is the quality and bias inherent in the data. Clinical datasets often suffer from incomplete records, inconsistent documentation, and noise, which can reduce the reliability of AI-based models [33]. Self-reported cases may lack precise details about the timing, dose, and the substances involved, introducing variability that compromises model performance. Additionally, bias in data collection—such as overrepresentation of certain demographic groups or geographic regions—can limit the generalizability of AI models [34]. For instance, a model trained predominantly on datasets from urban hospitals may underperform in rural or underserved settings where patterns of poisoning and health care infrastructure differ [35].

### Regulatory and Ethical Issues

The application of AI in emergency toxicology also raises regulatory and ethical concerns. Current regulations governing medical AI tools are evolving but remain inconsistent globally [36]. Validation, certification, and approval processes can be lengthy, potentially delaying the deployment of such tools. Ethical issues are also pronounced in this field, given the relative novelty of these tools. For example, AI models that make treatment recommendations could inadvertently exacerbate health inequities if trained on biased datasets. Additionally, explainability remains a critical issue, as many ML models operate as “black boxes,” making it difficult for clinicians to trust and act upon AI–driven predictions without a clear understanding of their rationale [37]. This will also impede regulatory acceptance.

### Clinician Trust and Usability

Clinician trust in AI applications involves the willingness of providers to depend on automated systems, reflecting the perceived reliability, accuracy, and relevance of AI recommendations [38]. Such trust must also be bidirectional, with clinicians trusting AI outputs, and systems designed in ways that trust human inputs [39]. AI solutions must thus demonstrate algorithmic transparency, robustness, and sound alignment with clinical reasoning, in order to establish trust between system and user [40,41].

Usability of AI tools also affects clinician adoption and sustained use. Poorly designed AI interfaces contribute to cognitive overload, impede rapid decision-making, and ultimately undermines trust; conversely, intuitive and explainable interfaces that integrate into clinical workflows facilitate acceptance [39].

### Implementation Barriers

There are also multiple practical barriers to deploying AI solutions in clinical settings. First, integration into existing electronic health record systems can be technically challenging and resource intensive. Second, clinicians may lack the training or confidence to use AI tools effectively, leading to underutilization [42]. Third, the real-time nature of emergency medicine demands rapid and reliable AI predictions, which may be hindered by inadequate computational infrastructure or connectivity issues at inference time in low-resource settings. Finally, cost constraints may limit access to advanced AI tools in settings where they are most needed.

### Necessity of AI Models

A critical question in the adoption of AI-based models in toxicology is whether their increased complexity is always necessary, or useful. Several studies included in this review demonstrated that simpler statistical methods, such as logistic regression, often match or even outperform more advanced ML algorithms in certain scenarios. For example, Behnoush et al [43] demonstrated that despite reasonable performance of ML models in predicting seizures in tramadol poisoning, a logistic regression model in fact had superior predictive performance with an equal number of important variables (AUC, 0.77 vs Naive-Bayes AUC, 0.71).

When compared against AI models, traditional regression models, given performance parity, have significant advantages that may render them preferable for certain applications. They are more computationally efficient and easily interpretable, and their simplicity allows clinicians to understand the contribution of individual predictors, fostering trust and facilitating integration into clinical workflows. Additionally, the scalability of AI and ML methods often comes with considerable resource requirements, including extensive computational infrastructure and large datasets. These prerequisites may not always be feasible in resource-constrained healthcare settings, limiting the practical application of such models. Regression models, on the other hand, can function effectively with smaller datasets and minimal computational demands, making them a robust option in many contexts.

While AI models offer undeniable potential in capturing complex, nonlinear interactions and high-dimensional patterns, their necessity should be evaluated on a case-by-case basis, and advanced methods should be used only when they demonstrably enhance predictive performance.

## Future Directions

AI in emergency clinical toxicology continues to develop as new data sources and technological capabilities expand. Looking ahead, two key areas hold significant promise to transform patient assessment and intervention: (1) integrating large-scale, heterogeneous datasets and (2) using data from wearable and Internet of Things (IoT) devices.

### Big Data Integration

Integration of large datasets from multiple sources holds promise for enhancing AI applications in emergency toxicology. Combining data from national databases, hospital electronic health records, and research databases can improve the robustness and accuracy of AI models [44]. Advanced data harmonization techniques and federated learning approaches can enable collaborative analysis, whilst maintaining data privacy [45]. By incorporating real-world evidence and longitudinal and time-series data, AI models could potentially evolve from static tools to dynamic systems capable of adapting to emerging trends and treatment strategies [46].

### Wearable and IoT Data

The proliferation of wearable devices has broadened the scope of real-time health monitoring, with direct implications for emergency clinical toxicology. These devices can continuously track vital signs, physical activity, biophysiometric parameters related to illicit drugs [47], and even real-time measurements of specific drug concentrations in the body [48], potentially detecting early signs of toxic exposure or overdose before the patient presents to the ED. As wearables become more sophisticated, AI algorithms can analyze the continuous data streams to detect anomalies, such as abrupt changes in heart rate or respiratory rate, that may suggest toxicity, thus enabling pre-emptive alerts. Further research is subsequently also necessary to demonstrate the cost-effectiveness and user-friendliness of these wearables and IoT solutions in day-to-day toxicology practice.

## Conclusions

The integration of AI in the emergency toxicology field brings about significant potential in improving diagnostic accuracy, patient outcomes and operational efficiency. While there have been promising advancements in the use of AI tools, implementation barriers such as regulatory and ethical considerations must be addressed to enhance its adoption in this field. Future research can also be done to explore the benefits of AI use in acute poisoning cases in terms of time and cost-savings in patient care. Additionally, conducting more prospective trials would be essential to build a robust evidence base to facilitate the use of AI in real-world clinical applications.

## Supplementary material

10.2196/73121 Multimedia Appendix 1 Summary of studies on artificial intelligence models in acute poisoning outcome prediction.

## Abbreviations

AI: artificial intelligence
AUC: area under the curve
ML: machine learning
NLP: natural language processing
NPDS: National Poison Data System
DNN: deep neural network
CCB: calcium channel blockers
ECG: electrocardiograph
DL: deep learning
RF: Random forest
XGB: Extreme Gradient Boost
SVM: Support Vector Machine
PSO: particle swarm optimization
EHR: electronic health record
IoT: Internet of Things
