# Supplementary material for: Artificial Intelligence Applications in Emergency Toxicology: Advancements and Challenges
Source: J Med Internet Res. 2025 Aug 22;27:e73121. doi: 10.2196/73121 (PMC12373298; doi:10.2196/73121)
Supplement: Multimedia Appendix 1 [file jmir-v27-e73121-s001.docx]

**Table S1: Summary of studies on AI models in acute poisoning outcome prediction**

| Publication | Poison | Variables Used | Outcome Measure | Number of Patients | Best Performing Model | Performance Statistics |
| --- | --- | --- | --- | --- | --- | --- |
| An AI algorithm for analyzing globus pallidus necrosis after carbon monoxide intoxication[1] | Carbon monoxide | Demographics, comorbidities, vital signs, standard laboratory tests, treatment | Globus pallidus necrosis | 261 | Random Forest | AUC: 0.64 |
| An AI algorithm for analyzing acetaminophen-associated toxic hepatitis[2] | Acetaminophen | Demographics, vital signs, standard laboratory tests, treatment | Toxic hepatitis | 187 | Random Forest | AUC: 0.98 |
| Machine learning algorithms to predict seizure due to acute tramadol poisoning[3] | Tramadol | Demographics, vital signs, standard laboratory tests, manner of poisoning, history of addiction | Seizure | 909 | Naive-Bayes* | AUC: 0.71 |
| Heart-Brain 346-7 Score: Mortality prediction for carbon monoxide poisoning[4] | Carbon monoxide | Altered mental status, chest pain, syncope, shortness of breath, fire exposure, motor vehicle exposure, carboxyhaemoglobin levels, sex, age and presence of cardiac complication | Mortality | 1273 | Logistic Regression | AUC: 0.70 |
| Machine-learning based prediction model for acute kidney injury induced by multiple wasp stings[5] | Wasp stings | Demographics, clinical symptoms at admission, time of visit to Emergency Department, number of sting wounds, SOFA score at 24 hours, APACHE-II score, standard laboratory tests, length of hospital stay, mortality | Acute kidney injury | 214 | Logistic Regression-based Nomogram | AUC: 0.757 |
| Interpretable ML for the prediction of death risk in patients with acute diquat poisoning[6] | Diquat | Demographics, shock index, standard laboratory tests, diquat dose | Mortality | 201 | Random Forest | AUC: 0.98 |
| Utility of support vector machine and decision tree to identify the prognosis of metformin poisoning[7] | Metformin | Demographic, purpose of exposure, chronicity, clinical features, vital signs, patient symptoms | Severity (minor, moderate, major symptoms) | 2878 | Support Vector Machine | AUC: Minor 0.90, Moderate 0.82, Major 0.98 |
| Outcome prediction of methadone poisoning in the United States[8] | Methadone | Demographics, route of exposure, formulation, reason of exposure, chronicity | Severity (minor, moderate, major symptoms) | 3847 | Extreme Gradient Boosting | AUC: Minor 0.90, Moderate 0.81, Major 0.91 |
| Prediction of acute methanol poisoning prognosis using machine learning techniques[9] | Methanol | Demographics, vital signs, comorbidities, visual symptoms at admission, mental status, antidote therapy, ingested dose, standard laboratory tests, ventilator use, length of stay | Severity | 897 | Gradient Boosting | AUC: 0.947 |
| The role of decision tree and machine learning models for outcome prediction of bupropion exposure[10] | Bupropion | Demographics, chronicity, reason of exposure, route of exposure, formulation, clinical features, vital signs, ECG, electrolyte levels | Severity (minor, moderate, major symptoms) | 14931 | Light Gradient Boosting, Random Forest | AUC: LGM Major 0.95, RF Major 0.95 |
| The value of machine learning for prognosis prediction of diphenhydramine exposure[11] | Diphenhydramine | Demographics, reason of exposure, chronicity, formulation, patient symptoms | Severity (minor, moderate, major symptoms) | 53761 | Random Forest | AUC: 0.85 |

This is a Multimedia Appendix to a full manuscript published in the J Med Internet Res. For full copyright and citation information see http://dx.doi.org/10.2196/jmir.73121

**References**

1. Chan M-J, Hu C-C, Huang W-H, Hsu C-W, Yen T-H, Weng C-H. An artificial intelligence algorithm for analyzing globus pallidus necrosis after carbon monoxide intoxication. Hum Exp Toxicol 2023 Jan;42:9603271231190906. PMID:37491827

2. Yen J-S, Hu C-C, Huang W-H, Hsu C-W, Yen T-H, Weng C-H. An artificial intelligence algorithm for analyzing acetaminophen-associated toxic hepatitis. Hum Exp Toxicol SAGE Publications; 2021 Nov;40(11):1947–1954. PMID:33955253

3. Behnoush B, Bazmi E, Nazari SH, Khodakarim S, Looha MA, Soori H. Machine learning algorithms to predict seizure due to acute tramadol poisoning. Hum Exp Toxicol SAGE Publications; 2021 Aug;40(8):1225–1233. PMID:33538187

4. Rose JJ, Zhang MS, Pan J, Gauthier MC, Pizon AF, Saul MI, Nouraie SM. Heart-Brain 346-7 Score: the development and validation of a simple mortality prediction score for carbon monoxide poisoning utilizing deep learning. Clin Toxicol (Phila) Informa UK Limited; 2023 Jul;61(7):492–499. PMID:37417305

5. Wu W, Zhang Y, Zhang Y, Qu X, Zhang Z, Zhang R. Machine-learning based prediction model for acute kidney injury induced by multiple wasp stings. Toxicon Elsevier BV; 2024 Nov 6;250(108112):108112. PMID:39349067

6. Li H, Liu Z, Sun W, Li T, Dong X. Interpretable machine learning for the prediction of death risk in patients with acute diquat poisoning. Sci Rep Springer Science and Business Media LLC; 2024 Jul 12;14(1):16101. PMID:38997450

7. Mehrpour O, Saeedi F, Hoyte C, Goss F, Shirazi FM. Utility of support vector machine and decision tree to identify the prognosis of metformin poisoning in the United States: analysis of National Poisoning Data System. BMC Pharmacol Toxicol Springer Science and Business Media LLC; 2022 Jul 13;23(1):49. PMID:35831909

8. Mehrpour O, Saeedi F, Vohra V, Hoyte C. Outcome prediction of methadone poisoning in the United States: implications of machine learning in the National Poison Data System (NPDS). Drug Chem Toxicol Informa UK Limited; 2024 Sep;47(5):556–563. PMID:37941394

9. Rahimi M, Hosseini SM, Mohtarami SA, Mostafazadeh B, Evini PET, Fathy M, Kazemi A, Khani S, Mortazavi SM, Soheili A, Vahabi SM, Shadnia S. Prediction of acute methanol poisoning prognosis using machine learning techniques. Toxicology Elsevier BV; 2024 May;504(153770):153770. PMID:38458534

10. Mehrpour O, Saeedi F, Vohra V, Abdollahi J, Shirazi FM, Goss F. The role of decision tree and machine learning models for outcome prediction of bupropion exposure: A nationwide analysis of more than 14 000 patients in the United States. Basic Clin Pharmacol Toxicol Wiley; 2023 Jul;133(1):98–110. PMID:36960587

11. Mehrpour O, Saeedi F, Abdollahi J, Amirabadizadeh A, Goss F. The value of machine learning for prognosis prediction of diphenhydramine exposure: National analysis of 50,000 patients in the United States. J Res Med Sci Medknow; 2023 Jun 12;28(1):49. PMID:37496638
